# Loss of alveolar membrane diffusing capacity and pulmonary capillary blood volume in pulmonary arterial hypertension

**DOI:** 10.1186/1465-9921-14-6

**Published:** 2013-01-22

**Authors:** Samar Farha, Daniel Laskowski, Deepa George, Margaret M Park, WH Wilson Tang, Raed A Dweik, Serpil C Erzurum

**Affiliations:** 1 Respiratory Institute, Cleveland Clinic, 9500 Euclid Avenue, Cleveland, OH, 44195, USA; 2 Department of Pathobiology, Lerner Research Institute, Cleveland Clinic, 9500 Euclid Avenue, Cleveland, OH, 44195, USA; 3 Heart and Vascular Institute, Cleveland Clinic, 9500 Euclid Avenue, Cleveland, OH, 44195, USA

**Keywords:** Membrane diffusion, Pulmonary arterial hypertension, Lung capillary blood volume

## Abstract

**Background:**

Reduced gas transfer in patients with pulmonary arterial hypertension (PAH) is traditionally attributed to remodeling and progressive loss of pulmonary arterial vasculature that results in decreased capillary blood volume available for gas exchange.

**Methods:**

We tested this hypothesis by determination of lung diffusing capacity (DL) and its components, the alveolar capillary membrane diffusing capacity (D_m_) and lung capillary blood volume (V_c_) in 28 individuals with PAH in comparison to 41 healthy individuals, and in 19 PAH patients over time. Using single breath simultaneous measure of diffusion of carbon monoxide (DL_CO_) and nitric oxide (DL_NO_), DL and D_m_ were respectively determined, and V_c_ calculated. D_m_ and V_c_ were evaluated over time in relation to standard clinical indicators of disease severity, including brain natriuretic peptide (BNP), 6-minute walk distance (6MWD) and right ventricular systolic pressure (RVSP) by echocardiography.

**Results:**

Both DL_CO_ and DL_NO_ were reduced in PAH as compared to controls and the lower DL in PAH was due to loss of both D_m_ and V_c_ (all p < 0.01). While DL_CO_ of PAH patients did not change over time, DL_NO_ decreased by 24 ml/min/mmHg/year (p = 0.01). Consequently, D_m_ decreased and V_c_ tended to increase over time, which led to deterioration of the D_m_/V_c_ ratio, a measure of alveolar-capillary membrane functional efficiency without changes in clinical markers.

**Conclusions:**

The findings indicate that lower than normal gas transfer in PAH is due to loss of both D_m_ and V_c_, but that deterioration of D_m_/V_c_ over time is related to worsening membrane diffusion.

## Background

Lung diffusing capacity for carbon monoxide (DL_CO_) is a valuable clinical tool in the assessment of pulmonary diseases. It measures the ability of the lungs to transfer gas from the alveolar space to the red blood cells in the pulmonary vessels. Measurement of DL is informative for pathophysiologic diagnoses of lung diseases and serial measurements are used to follow the course of disease [1]. The components of DL can provide more detailed knowledge of the mechanisms of loss of gas exchange capacity. Based on the Roughton and Forster model [2], DL_CO_ is described as a series of resistances: the diffusion of the gas across the alveolar-capillary membrane, the transfer into the plasma and across the red blood cell membrane, and the chemical reaction of the gas with hemoglobin (Hgb). The following equation summarizes these concepts:

(1) $${1/\mathrm{DL}=1/\text{D}}_{\text{m}}{+1/\mathrm{\theta V}}_{c}$$

Where DL represents total lung diffusing capacity; D_m_ alveolar-capillary membrane diffusing capacity; V_c_ pulmonary capillary bed available for gas transfer; and θ is the rate of reaction of the gas with the red cell. Traditionally, measure of the two components of DL_CO_ relied upon repeated performance of the test at different oxygen concentrations and solving the equation for D_m_ and V_c_. These technical challenges resulted in an arduous method and inaccuracies in measures that limited utility. However, nitric oxide (NO) has a greater affinity to Hgb compared to CO or O_2_, and thus NO diffusion is mainly limited by the transfer of gas across the alveolar capillary membrane [3]. Based on this, DL_NO_ can be used as a direct measure of D_m_. Recent advances allow measure of DL_NO_ and DL_CO_ simultaneously and direct determination of D_m_ and V_c_ from single breath maneuver at one oxygen tension [3,4]. Few studies have evaluated the components of DL in pulmonary hypertension (PH) in an attempt to understand the pathophysiology of the decreased DL. Cross-sectional data from studies are inconsistent and there are no longitudinal data. In 1968, Nadel et al. showed that DL_CO_ was reduced in PH as a result of low V_c _[5]. Borland et al. were the first to apply a single breath technique to measure DL_NO_ and DL_CO_ and identified that both were reduced in PH [6]. However, recent studies have attributed the low DL to a loss of D_m_ rather than V_c _[7,8]. Oppenhiemer et al. measured D_m_ and V_c_ in different groups of PH patients and found that D_m_ was reduced out of proportion to V_c_ as shown by a reduction in the ratio of D_m_/V_c_[9]. Measuring the components of DL in PAH would give insight into the disease, especially that recent advances have unveiled the intricacy of the pathogenesis of PAH. We hypothesized that both D_m_ and V_c_ are decreased in PAH patients and that there is progressive decline of DL and its components over time in parallel to disease progression. To test this, DL_NO_ and DL_CO_ were measured by single breath technique in PAH patients in comparison to healthy controls and over time in order to evaluate changes in D_m_ and V_c_ in relation to clinical parameters including echocardiography, 6MWD and BNP levels.

## Materials and methods

41 healthy controls and 28 subjects with PAH were enrolled in the study. The Institutional Review Board at the Cleveland Clinic approved the study and all subjects gave written informed consent (IRB 7853). Healthy controls were recruited from the community, had no medical problems and were on no medications. PAH subjects were recruited from the PH clinic. They had an established diagnosis of PAH based on right heart catheterization. Their medical records were reviewed to verify diagnosis. Clinical data including 6MWD, BNP, complete cell count and metabolic panel were collected from the medical records when available. Lung function testing including spirometry, lung volumes, DL_CO_ and DL_NO_ as well as exhaled NO and echocardiogram were measured as part of the study. These tests were repeated longitudinally for a subgroup of PAH patients.

### Exhaled NO, lung function, volumes and diffusing capacity for carbon monoxide and nitric oxide

Single-breath on-line measurement of fractional NO concentration in expired breath (FE_NO_) was measured at the beginning of each visit using the NIOX (Aerocrine, NJ) [10].

The forced expiratory volume at 1 second (FEV_1_) and vital capacity (FVC) were measured by spirometry (MasterLab; Viasys/Jaeger; Höchberg, Germany) following established guidelines [11]. Total lung capacity (TLC) was measured using the single breath measurement method [12]. Helium was used as a tracer gas following the ATS/ERS guidelines.

Single breath measurements of DL_CO_ and DL_NO_ were performed using a modified Masterscreen PFT (Viasys/Jaeger; Höchberg, Germany) adapted to measure nitric oxide. The measurement was performed with the patient rested in a seated position using a nose clip. The inspired gas contained 70 ppm nitric oxide (Ikara, NJ) and a blend of ultra high purity 21% oxygen, 0.28% carbon monoxide, 9.5% helium and balance nitrogen. The single breath DL_CO_ method was performed in duplicate to a maximum of 4 measurements to obtain 2 measurements within 5%, ~4 minutes apart using a washout volume of 750 ml and an alveolar volume of 750 ml per standard guidelines [1,13]. The breathhold time was ~6 seconds [13]. The instrument was calibrated daily. D_mCO_ was calculated as DL_NO_ divided by 1.97 based on solubility factors for CO and NO respectively of 0.0183 and 0.0364 [14].

V_c_ was calculated from the following formula [2]:

(2) $$\begin{array}{ll} 1/V_{c} & =\left(1/{\mathrm{DL}}_{\mathrm{CO}}–1/D_{\mathrm{mCO}}\right)\times \theta_{\mathrm{CO}},\mathrm{where}\;1/\theta_{\mathrm{CO}}\\ & =\left(0.73+0.0058\times P_{\mathrm{AO}2}\right)\times 14.6/\mathrm{Hb}=1.474 \end{array}$$

(3) $${\text{P}}_{\text{AO}2}={\text{F}}_{\text{AO}2}\times \left(\mathrm{Patm}-47\right)\mathrm{and}\;\mathrm{Hb}=14.6$$

### Echocardiogram

Two dimensional echocardiograms and doppler exams were performed on the same day as the DL test or in some cases (mainly for the first visit) within up to 8 weeks of the DL test for convenience of the participant.

### Echocardiographic analysis

All echocardiographic analysis were performed following the American Society of Echocardiography Guidelines and Standards [15].

From the parasternal long axis view the Interventricular septal (IVS) thickness in end-diastole, left ventricular end-diastolic dimension (LVEDD), left ventricular end-systolic dimension (LVESD) and posterior wall thickness in diastole were measured from the 2D parasternal long axis image following ASE guidelines. Left ventricular (LV) mass was determined from 2D measurements using the cubed formula:

(4) $$\begin{array}{ll} \mathrm{LV}\;\mathrm{Mass}= & 1.04\left({\left(\mathrm{IVS}+\mathrm{PW}+\mathrm{LVEDD}\right)}^{3}-{\mathrm{LVEDD}}^{3}\right)\\ & –13.6\text{g}\left(\mathrm{Devereaux}\;\mathrm{Regression}\right) \end{array}$$

LV function: LV ejection fraction was determined by visual assessment, and/or apical biplane volumes. LV end-diastolic and end-systolic volumes were calculated from the apical 4 and 2 chamber views using the modified Simpson method. LV fractional shortening was determined from parasternal 2D analysis as [(LVEDD-LVESD)/LVEDD] × 100.

Right ventricular (RV) function: RV end-diastolic area (RVAD) and end-systolic area (RVAS) were measured in the apical 4-chamber view by tracing the endocardial border of the RV and the tricuspid annular plane. RV fractional area change was calculated as follows:

(5) $$\begin{array}{ll} \mathrm{RV}\;\mathrm{fractional}\;\mathrm{area}= & \left[\left(\mathrm{RVAD-RVAS}\right)/\mathrm{RVAD}\right]\\ & \times 100 \end{array}$$

Right atrial volume was measured in the apical 4 chamber view by using the single plane area length method.

The right ventricular systolic pressure (RVSP) was estimated from the systolic pressure gradient between the RV and the right atrium by the peak continuous-wave Doppler velocity of the TR jet using the modified Bernoulli equation plus estimated right atrial pressure (RAP). RAP was estimated from the subcostal window approach measuring changes in inferior vena caval size and collapsibility as determined by the respiratory sniff test following ASE guidelines.

Echo-Doppler estimation of pulmonary vascular resistance (PVR): The highest Doppler continuous wave tricuspid valve peak velocity jet obtained from multiple views (parasternal long axis, parasternal short axis, apical 4 chamber, subcostal or apical off-axis imaging) was determined as the maximum tricuspid regurgitant velocity (TRV). Pulsed wave Doppler sample was placed in the right ventricular outflow tract (RVOT) at the level of the aortic valve in the parasternal short axis view just below the pulmonic valve so that pulmonic valve closure is identified. The Doppler spectrum was traced to determine the time velocity integral of the RVOT (RVOT-TVI). PVR calculation was determined by:

(6) $$\begin{array}{ll} \mathrm{PVR}\left(\mathrm{Wood}\;\mathrm{units}\right)= & 10\times \mathrm{TRV}\left(\text{m}/\sec\right)/\mathrm{RVOT}\\ & -\mathrm{TVI}\left(\mathrm{cm}\right)+.16 \end{array}$$

### Statistics

For PAH patients, individual patient slopes representing changes over actual time were calculated using linear regression for each continuous study variable. For individual variables, Wilcoxon signed rank tests were used to test the null hypotheses of zero mean slopes for the PAH patients. The 2 groups were compared with respect to baseline variables using Fisher's exact and chi-square tests for categorical variables, and Kruskal-Wallis and Wilcoxon rank sum tests for quantitative variables. Spearman correlation coefficients measured at specified time points, were used to describe relationships among pairs of quantitative variables in a manner free of the normality assumption. All analyses were performd using R version 2.4.1. (R Development Core Team (2011). R Foundation for Statistical Computing, Vienna, Austria. ISBN 3-900051-07-0, URL http://www.R-project.org/).

## Results

Patients were classified according to Dana Point classification of pulmonary hypertension: Class 1.1 (n = 21) 75%, Class 1.2 (n = 3) 10.7%, Class 1.3 (n = 1) 3.6% and Class 1.4 (n = 3) 10.7%. Three patients were Class 1.4 and respectively had systemic sclerosis, lupus and chronic hemolytic anemia. None of the PAH patients had interstitial lung disease or obstructive lung disease based on clinical history, exam, computerized chest tomography imaging, as part of the evaluation and assignment of PAH class. One patient had mild pulmonary edema on chest X-ray. Height, weight and gender distribution was similar among PAH patients and controls; however, PAH patients were older (p < 0.01). Table 1 shows the baseline characteristics of the two groups and Table 2 lists the clinical data of the PAH patients. The study spanned 15 months with a mean time of 5 months between visit 1 and visit 2 and of 4 months between visit 2 and visit 3. Twenty-eight PAH patients were evaluated at baseline, of those 19 elected to continue with the longitudinal study and were followed over time. Fourteen patients completed all 3 visits. There was no difference in baseline characteristics between the patients who were followed longitudinally and those who were not. The patients followed longitudinally had mainly idiopathic PAH [Class 1.1 (N = 15) 79%, Class 1.2 (N = 2) 11%, Class 1.3 (N = 1) 5% and Class 1.4 (N = 1) 5%]. During the time span of the study, none of the PAH patients developed new findings on chest imaging performed as part of their clinical follow up.

**Table 1:** Baseline characteristics of the two study groups

**Variable**	**Healthy**	**PAH**	**p**-**value**
	**N = 41**	**N = 28**	
Age (years)	34 ±2	45 ±2	<0.01
Gender (M/F)	17/24	6/22	0.09
Smoking history (never/current/ex smoker)	40/0/1	24/1/3	0.1
Height (cm)	169 ±1	167 ±3	0.3
Weight (kg)	79 ±3	89 ±7	0.2
O_2_ Saturation (% of Hgb)	98 ±0.2	96 ±0.4	<0.01
FE_NO_ (ppb)	19 ±2	18 ±2	0.7
FVC (%)	98 ±2	91 ±3	0.2
FEV_1_ (%)	93 ±2	80 ±4	<0.01
FEV_1_/FVC	81 ±1	75 ±1	<0.01
TLC (%)	85 ±2	82 ±2	0.4
V_A_ (L)	4.9 ±0.2	4.3 ±0.2	0.1
DL_CO_ (ml/min/mmHg)	24 ±1	17 ±1	<0.01
DL_NO_ (ml/min/mmHg)	94 ±4	66 ±5	<0.01
D_m_ (ml/min/mmHg)	48 ±2	33 ±2	<0.01
V_c_ (ml)	78 ±4	63 ±5	<0.01
D_m_/V_c_ (1/min/mmHg)	0.6 ±0.02	0.6 ±0.04	0.8
Hgb (g/dl)	13.3 ±0.3	13.5 ±0.4	0.9

F/M: female/male.

FE_NO_: fractional exhaled nitric oxide.

FVC: forced vital capacity.

FEV_1_: forced expiratory volume in one second.

TLC: total lung capacity.

V_A_: alveolar volume.

DL: lung diffusion for carbon monoxide (DL_CO_) and for nitric oxide (DL_NO_).

D_m_: alveolar-capillary membrane diffusing capacity.

V_c_: lung capillary blood volume.

Hgb: hemoglobin.

PAH: pulmonary arterial hypertension.

**Table 2:** Clinical data of PAH group

**Etiology of PAH n(%)**	
Idiopathic	21(75%)
Heritable	3(10.7%)
Drug and toxin-induced	1(3.6%)
Associated with	3(10.7%)
NYHA class n(%)	
I	4(14%)
II	14(50%)
III	10(36%)
IV	0(0%)
Medications n(%)	
Prostacyclin	6(21.4%)
PDE5i	4(14.3%)
ERA	4(14.3%)
Prostacyclin + PDE5i	4(14.3%)
Prsoatcyclin + ERA	2(7.15%)
ERA + PDE5i	2(7.15%)
Prostacyclin + PDE5i + ERA	6(21.4%)
6-MWD (m)	487±24
Right ventricular systolic pressure (mmHg)	78 ±5
Mean pulmonary arterial pressure (mmHg)	52±3
Mean pulmonary capillary wedge pressure (mmHg)	13±1
Cardiac output (L/min)	6±0.4
Cardiac index (L/min/m^2^)	3±0.2
Pulmonary vascular resistance (wood units)	7.8±0.9

PAH: pulmonary arterial hypertension.

NYHA: new york heart association.

PDE5i: phosphodiesterase type 5 inhibitor.

ERA: endothelin receptor antagonist.

6-MWD: 6 minute walk distance.

### Airflow and diffusion capacity

Oxygen saturation at rest was lower in PAH patients compared to controls [Table 1. PAH patients had reduced airflow as compared to controls [Table 1. As previously reported in PAH patients treated with prostacyclins [16,17], exhaled NO of patients was similar to controls [Table 1. DL measured by CO or NO was lower in PAH as in previous reports [Table 1. The alveolar volume was not different in PAH compared to controls [Table 1. DL corrected for V_A_ was reduced in PAH compared to controls [DL_CO_/V_A_ (1/min/mmHg): PAH 3.9 ± 0.1, controls 5 ± 0.1; p < 0.01, DL_NO_/V_A_ (1/min/mmHg): PAH 14.9 ± 0.7, controls 19.3 ± 0.4; p < 0.01]. Similarly, DL corrected for Hgb was lower in PAH [DL_CO_ corrected for Hgb (ml/min/mmHg): PAH 17.3 ± 1.0, controls 23.6 ± 1.3; p < 0.01, DL_NO_ corrected for Hgb (ml/min/mmHg): PAH 65.5 ± 4.5, controls 90.4 ± 5.0; p < 0.01]. Both D_m_ and V_c_ were reduced in PAH [Table 1. To delineate whether proportionate loss was accountable for the lower DL in PAH, the ratio of D_m_/V_c_ was calculated. The ratio was similar between the two groups, suggesting a proportionate loss of D_m_ and V_c_ in PAH [Table 1. Mean variabilities of DL_CO_ and DL_NO_ for healthy controls were 3% and 5% respectively and for PAH 4% and 7%.

### D_m_ and V_c_ in subjects with PAH over time

**Figure 1:**
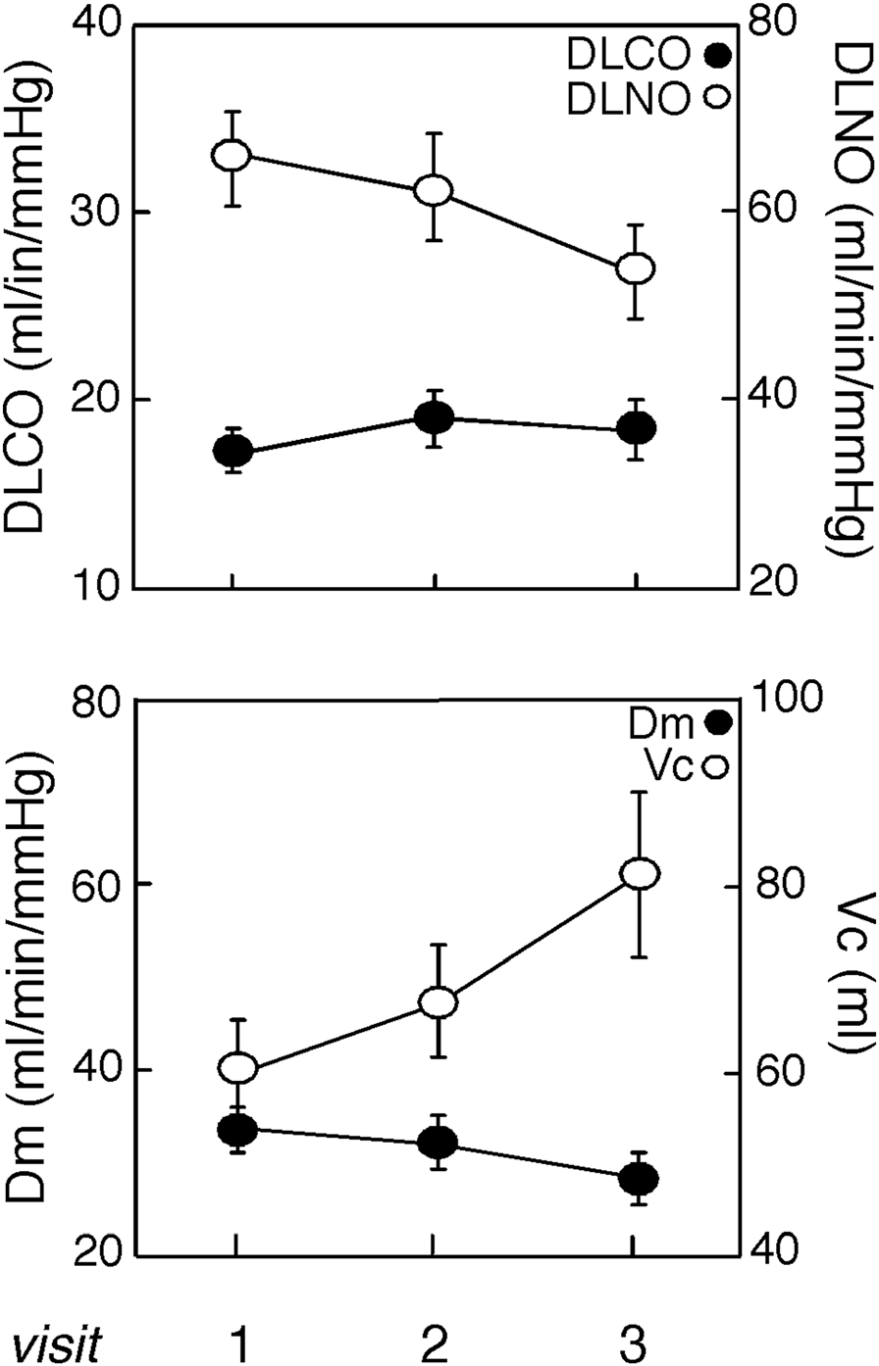
**Lung diffusion capacity and its components over time in pulmonary arterial hypertension.** Lung diffusing capacity for NO (DLNO) and the alveolar-capillary membrane diffusing capacity (D_m_) decreases over time (both p = 0.01). Lung capillary blood volume (V_c_) tends to increase over time (p = 0.07). Conversely, DLCO did not change significantly (p = 0.7).

Although DL_CO_ was stable over time [median DL_CO_ = −1.2 ml/min/mmHg/year (IQR −2.8, 1.5), Wilcoxon SR p = 0.7], there was a significant decline in DL_NO_ and hence D_m_ [median DL_NO_ = −23.8 ml/min/mmHg/year (IQR −36.8, -9.9), Wilcoxon SR p = 0.01] [median D_m_ = −11.9 ml/min/mmHg/year (IQR −18.5, -4.3), Wilcoxon SR p = 0.01] (Figure 1) [Table 3]. On the other hand, V_c_ tended to increase over time, accounting for stability of DL_CO_ [median V_c_ = 10.9 ml/year (IQR 4.2, 46.9), Wilcoxon SR p = 0.07] (Figure 1). The ratio of D_m_/V_c_ decreased over time, which indicated a disproportionate loss of membrane diffusion compared to capillary bed [median D_m_/V_c_ = −0.4 1/min/mmHg/year (IQR −0.5, -0.05), Wilcoxon SR p = 0.04] [Table 3]. Hgb did not vary over time [Table 3]. Likewise, DL_CO_ corrected for Hgb did not vary significantly (Wilcoxon SR p = 0.3) whereas DL_NO_ corrected for Hgb decreased (Wilcoxon SR p < 0.01). The alveolar volume did not change over time (Wilcoxon SR p = 0.4) [Table 3].

**Table 3:** Changes over time in the PAH group

**Variable**	**Estimated unit/year change**	**Wilcoxon SR p**-**value**
FEV_1_ (ml)	−144	0.1
FEV_1_ (%)	−3	0.12
FVC (ml)	−190	0.2
FVC (%)	−5	0.2
FEV_1_/FVC	0.8	0.4
V_A_ (ml)	20	0.4
FE_NO_ (ppb)	0.5	0.4
TLC (%)	0.7	0.5
DL_CO_ (ml/min/mmHg)	−1.2	0.7
DL_NO_ (ml/min/mmHg)	−24	0.01
D_m_ (ml/min/mmHg)	−12	0.01
V_c_ (ml)	11	0.07
D_m_/V_c_ (1/min/mmHg)	−0.4	0.04
Hgb (g/dl)	0.05	0.6

FE_NO_: fractional exhaled nitric oxide.

FVC: forced vital capacity.

FEV_1_: forced expiratory volume in one second.

TLC: total lung capacity.

Va: alveolar volume.

DL: lung diffusion for carbon monoxide (DL_CO_) and for nitric oxide (DL_NO_).

D_m_: alveolar-capillary membrane diffusing capacity.

V_c_: lung capillary blood volume.

Hgb: hemoglobin.

The rest of the lung functions measured and exhaled NO did not vary (all Wilcoxon SR p > 0.05).

### Changes in clinical parameters over time

**Figure 2:**
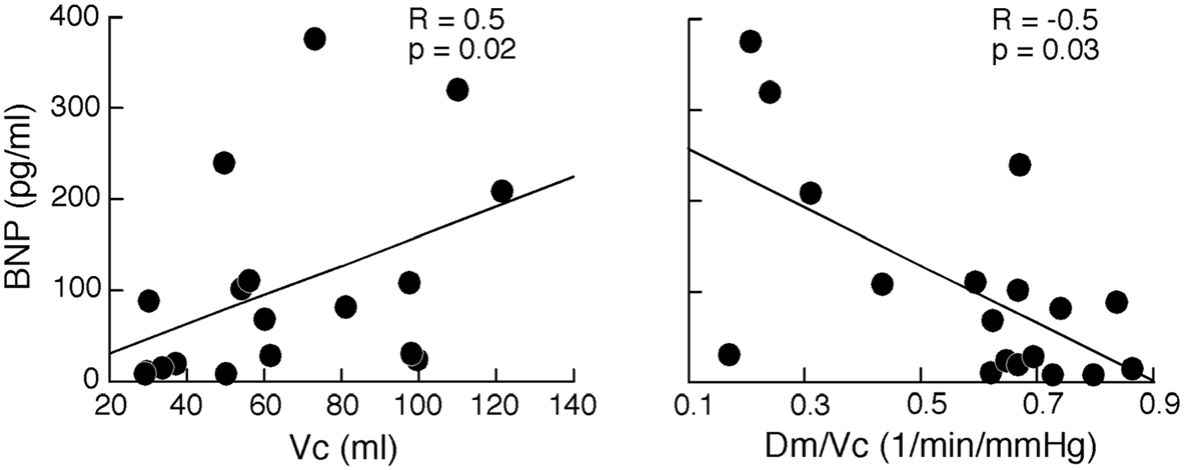
**Association of brain natriuretic peptide (BNP) to lung capillary blood volume (V**_**c**_**) and to the ratio of membrane diffusing capacity (D**_**m**_**) and V**_**c**_**.** D_m_/V_c_ is inversely related to disease severity measured by BNP whereas V_c_ correlates directly to BNP. The points represent measurements obtained at time of first visit.

Baseline clinical data for the PAH patients are summarized in Table 2. Right heart catheterization data were retrieved from the medical records and the most recent one was used. Some (N = 15) were performed within the same year, however others were performed in past years. Clinical parameters including echocardiogram, 6MWD and BNP, did not decline significantly over time of the study. At baseline, DL_NO_ and D_m_ did not correlate with clinical markers [Table 4]. BNP was associated with V_c_ and D_m_/ V_c_ (V_c_: Spearman R = 0.5, p = 0.02; D_m_/ V_c_: Spearman R = −0.5, p = 0.03) (Figure 2) at visit 1 but not at visit 2 or 3 (p > 0.1). In addition, V_c_ was inversely related to RV function (RVAD: Spearman R = 0.4, p = 0.05, RVAS: Spearman R = 0.4, p = 0.04) suggesting that V_c_ increases with worsening PAH/RV function. V_c_ and RV fractional area change did not correlate significantly (Spearman R = −0.4, p = 0.09). DL and its components did not correlate with left ventricular systolic and diastolic function (all p > 0.1). DL_CO_ correlated with the 6MWD (Spearman R = 0.5, p = 0.05) [Table 4].

**Table 4:** Correlations among clinical markers and lung diffusion capacity and its components at baseline

**Variables**	**DL**_**CO**_**(ml/min/mmHg)**	**DL**_**NO**_**(ml/min/mmHg)**	**D**_**m**_**(ml/min/mmHg)**	**V**_**c**_**(ml)**
BNP (pg/ml)	R = 0.3	R = −0.01	R = −0.01	R = 0.5
	P = 0.2	P = 0.9	P = 0.9	p = 0.02
6-MWD (m)	R = 0.5	R = 0.4	R = 0.4	R = 0.3
	p = 0.05	p = 0.1	p = 0.1	P = 0.2
Left ventricular fractional shortening (%)	R = −0.3	R = −0.3	R = −0.3	R = −0.3
	p = 0.1	p = 0.2	p = 0.2	p = 0.3
Left ventricular ejection fraction (%)	R = −0.1	R = −0.1	R = −0.1	R = −0.2
	p = 0.6	p = 0.6	p = 0.6	p = 0.4
Right ventricular systolic pressure (mmHg)	R = 0.09	R = −0.1	R = −0.1	R = 0.3
	p = 0.7	p = 0.6	p = 0.6	p =0.2
Right ventricular end diastolic area (cm^2^)	R = 0.2	R = 0.07	R = 0.07	R = 0.4
	p = 0.3	p = 0.7	p = 0.7	p = 0.04
Right ventricular end systolic area (cm^2^)	R = 0.2	R = 0.07	R = 0.07	R = 0.4
	p = 0.4	p = 0.8	p = 0.8	p = 0.04
Right ventricular fractional area change (%)	R = −0.2	R = −0.2	R = −0.2	R = −0.3
	p = 0.3	p = 0.4	p = 0.4	p = 0.09
RV fractional shortening (%)	R = −0.2	R = −0.2	R = −0.2	R = −0.3
	p = 0.4	p = 0.4	p = 0.4	p = 0.1
Right ventricular outflow tract velocity time interval (cm)	R = −0.3	R = −0.2	R = −0.2	R = −0.1
	p = 0.2	p = 0.4	p = 0.4	p = 0.6
Mean pulmonary arterial pressure (mmHg)	R = 0.2	R = 0.06	R = 0.06	R = 0.4
	p = 0.3	p = 0.8	p = 0.8	p = 0.05
Mean pulmonary capillary wedge pressure (mmHg)	R = 0.2	R = −0.05	R = −0.05	R = 0.2
	p = 0.5	p = 0.8	p = 0.8	p = 0.4
Cardiac output (L/min)	R = −0.3	R = −0.4	R = −0.4	R = −0.2
	p = 0.3	p = 0.1	p = 0.1	p = 0.5
Pulmonary vascular resistance (wood units)	R = 0.2	R = 0.2	R = 0.2	R = 0.3
	p = 0.4	p = 0.4	p = 0.4	p = 0.1

6-MWD: 6 minute walk distance.

BNP: brain natriuretic peptide.

DL: lung diffusion for carbon monoxide (DL_CO_) and for nitric oxide (DL_NO_).

D_m_: alveolar-capillary membrane diffusing capacity.

V_c_: lung capillary blood volume.

## Discussion

This is the first longitudinal study to describe the changes in gas transfer in PAH over time. Lung diffusing capacity for NO, but not CO, dropped due to the disproportionate loss of membrane diffusion, Dm, as compared to changes in the vascular bed available for gas exchange, Vc. These findings suggest that the efficiency of the alveolar-capillary unit in PAH worsens over time independent of traditional clinical and echocardiographic measures. The results also put forward the potential utility of DL_NO_ in tracking progression of disease in PAH.

As in prior work, DL was reduced in PAH patients. Although PAH patients and controls were not well matched by age and gender, the decrease in DL is not likely explained by age and gender alone. Earlier studies measured DL_CO_ at two different oxygen concentrations and calculated D_m_ and V_c_ except for the study by Borland et al. where the single breath test measuring both CO and NO uptake was used. All discovered a decrease in DL, but earlier studies showed predominant loss of V_c_ while more recent ones showed that D_m_ is reduced out of proportion to V_c_. The difference between earlier studies and more recent ones could be attributed to differences in techniques or use of pulmonary vasodilators leading to higher measured V_c_. In this study, the single breath technique measuring DL_NO_ and DL_CO_ was used to assess D_m_ and V_c_. D_m_ was reduced proportionally to V_c_ in patients with PAH, and thus D_m_/V_c_ was not different from controls. This confirms the interdependence of D_m_ and V_c_ in pulmonary vascular diseases and any change in the vascular bed that reduces V_c_ would lead to a reduction in D_m_ through a reduction in the surface area available to gas exchange.

In PAH, the reduction in V_c_ is likely multifactorial: increased pulmonary vascular resistance, decreased cardiac output and local thrombosis of the vascular bed. Mechanisms underlying the reduced D_m_ may include an increase in the alveolar-capillary membrane thickness caused by fibrotic or proliferative process, and/or interstitial edema. Of note, none of the patients recruited for the study had interstitial lung disease. The decreased DL in association with decreased D_m_ and V_c_ has been described in chronic heart failure patients in stable clinical condition [18]. There is growing evidence that as disease progresses; the left ventricular function may become independently compromised in PAH [19,20]. This may contribute to the reduced DL, D_m_ and V_c_.

Over time, the increase in V_c_ in association with a decrease in D_m_ could be explained by the effect of vasodilator therapy on the diseased pulmonary vasculature. D_m_ would not be expected to increase proportionally to V_c_ with vasodilation alone as the thickened alveolar-capillary membrane is not affected and the fibroproliferative process is not responsive to pulmonary vasodilators. Another explanation could be worsening left side function with increase pulmonary capillary pressure and congestion associated with interstitial edema. In fact, similar findings are noted in heart failure, in which acute decompensation and increased wedge pressure cause a drop in D_m_ paralleled by an increase in V_c_[21]. Worsening RV function in PAH can lead to LV dysfunction through ventricular interdependence. Our findings showed that V_c_ increased with worsening RV function but there was no relation between V_c_ and LV function. In view of the limitations of echocardiography and the absence of repeat right heart catheterization, the contribution of LV dysfunction to the increase of V_c_ noted over time remains undetermined. Overall, the lack of perceptible changes in standard clinical markers highlights the limitations of available tests, and suggests the possibility that lung diffusing capacity as measured by NO, i.e. the lung D_m_, may be a potential marker for disease progression in PAH. A limitation of the study is the small number of patients followed longitudinally and studies with a large cohort of PAH patients over time in correlation with clinical parameters and outcomes are needed to confirm our findings.

Evidence of airway obstruction in PAH is suggested in this study based on the decreased %FEV_1_ and FEV_1_/FVC. This is in keeping with published data that identifies peripheral airway obstruction in PAH by lung functions and pathologic findings [22-26]. Inflammation typically surrounds plexiform lesions in PAH [27] and abundant mast cells have been noted in PAH lungs [28]. Lung biopsies show small airways narrowing with thickened walls infiltrated by lymphocytes, plasma cells and polymorphonuclear leukocytes [29]. Moreover, studies reveal shared mechanistic features of asthma and PAH in experimental models [30,31]. Thus, although PAH is defined as a pure vascular disease, impaired airflow occurs in patients on PAH therapies, suggesting a potential role for airway-directed therapies in the care of these patients.

There are several limitations to the study. Patients were recruited from our PH clinic and the research testing was done on the day of their scheduled clinical visits. Their primary physician scheduled follow up visits and adjusted PAH therapy. We had no control on the time interval between visits or on treatment. As such though most patients were stable on their PAH therapies, the effect of medications on the findings can not be excluded. Another limitation is the difference in age between the PAH and control groups. However, the novel finding here is the longitudinal changes in the PAH group. The drop in D_m_ over time has not been described previously. Another limitation is the lack of concurrent hemodynamic data at the time of the DL measurements. Future studies evaluating D_m_ and V_c_ in relation to hemodynamic data are essential to better understand the significance of the changes noted here.

## Conclusion

In summary, a reduction in both D_m_ and V_c_ was noted in PAH patients in association with highly efficient alveolar-capillary units. However, over time, there was a drop in D_m_/V_c_. Although the present study does not fully unveil the clinical importance of the decline in D_m_/V_c_, it opens the field to further investigate the single breath measurement of DL_NO_/DL_CO_ and its components in PAH.

## Abbreviations

BNP: Brain natriuretic peptide; CO: Carbon monoxide; DL: Lung diffusion for carbon monoxide; DL_CO_: Lung diffusion for carbon monoxide; DL_NO_: Lung diffusion for nitric oxide; D_m_: Alveolar-capillary membrane diffusing capacity; F/M: Female/male; FE_NO_: Fractional exhaled nitric oxide; FEV_1_: Forced expiratory volume in one second; FVC: Forced vital capacity; Hgb: Hemoglobin; IVS: Interventricular Septal thickness; LVEDD: Left Ventricular end diastolic diameter; LVESD: Left Ventricular end systolic diameter; NO: Nitric oxide; O_2_: Oxygen; PAH: Pulmonary arterial hypertension; PH: Pulmonary hypertension; PVR: Pulmonary vascular resistance; PW: Posterior wall thickness; RV: Right ventricle; RVAD: Right ventricular end-diastolic area; RVAS: Right ventricular end-systolic area; RVOT-TVI: Right ventricular outflow tract-time velocity integral; RVSP: Right ventricular systolic pressure; TLC: Total lung capacity; TRV: Tricuspid regurgitant velocity; V_A_: Alveolar volume; V_c_: Lung capillary blood volume; 6MWD: 6-minute walk distance.

## Competing interests

The authors declare that they have no competing interests.

## Authors’ contributions

SF conducted the study, performed research, analyzed and interpreted data and wrote the manuscript, DL performed research, DG conducted the study and recruited subjects, MMP conducted research and recruited subjects, WHWT analyzed data and reviewed manuscript, RAD reviewed manuscript, SCE designed research, analyzed data and wrote the manuscript. All authors participated in the study and have seen and approved the final version.

## References

[B1] Macintyre N Crapo RO Viegi G Johnson DC van der Grinten CP Brusasco V Burgos F Casaburi R Coates A Enright P Standardisation of the single-breath determination of carbon monoxide uptake in the lung Eur Respir J 2005 26 4 720 735 10.1183/09031936.05.00034905 16204605

[B2] Roughton FJ Forster RE Relative importance of diffusion and chemical reaction rates in determining rate of exchange of gases in the human lung, with special reference to true diffusing capacity of pulmonary membrane and volume of blood in the lung capillaries J Appl Physiol 1957 11 2 290 302 13475180 10.1152/jappl.1957.11.2.290

[B3] Guenard H Varene N Vaida P Determination of lung capillary blood volume and membrane diffusing capacity in man by the measurements of NO and CO transfer Respir Physiol 1987 70 1 113 120 10.1016/S0034-5687(87)80036-1 3659606

[B4] Borland CD Higenbottam TW A simultaneous single breath measurement of pulmonary diffusing capacity with nitric oxide and carbon monoxide Eur Respir J 1989 2 1 56 63 2707403

[B5] Nadel JA Gold WM Burgess JH Early diagnosis of chronic pulmonary vascular obstruction. Value of pulmonary function tests Am J Med 1968 44 1 16 25 10.1016/0002-9343(68)90233-7 5635285

[B6] Borland C Cox Y Higenbottam T Reduction of pulmonary capillary blood volume in patients with severe unexplained pulmonary hypertension Thorax 1996 51 8 855 856 10.1136/thx.51.8.855 8795679 PMC472573

[B7] Bernstein RJ Ford RL Clausen JL Moser KM Membrane diffusion and capillary blood volume in chronic thromboembolic pulmonary hypertension Chest 1996 110 6 1430 1436 10.1378/chest.110.6.1430 8989056

[B8] Steenhuis LH Groen HJ Koeter GH van der Mark TW Diffusion capacity and haemodynamics in primary and chronic thromboembolic pulmonary hypertension Eur Respir J 2000 16 2 276 281 10.1034/j.1399-3003.2000.16b15.x 10968503

[B9] Oppenheimer BW Berger KI Hadjiangelis NP Norman RG Rapoport DM Goldring RM Membrane diffusion in diseases of the pulmonary vasculature Respir Med 2006 100 7 1247 1253 10.1016/j.rmed.2005.10.015 16376536

[B10] ATS/ERS recommendations for standardized procedures for the online and offline measurement of exhaled lower respiratory nitric oxide and nasal nitric oxide, 2005 Am J Respir Crit Care Med 2005 171 8 912 930 15817806 10.1164/rccm.200406-710ST

[B11] Miller MR Hankinson J Brusasco V Burgos F Casaburi R Coates A Crapo R Enright P van der Grinten CP Gustafsson P Standardisation of spirometry Eur Respir J 2005 26 2 319 338 10.1183/09031936.05.00034805 16055882

[B12] Wanger J Clausen JL Coates A Pedersen OF Brusasco V Burgos F Casaburi R Crapo R Enright P van der Grinten CP Standardisation of the measurement of lung volumes Eur Respir J 2005 26 3 511 522 10.1183/09031936.05.00035005 16135736

[B13] Cotes J Lung Function 1993 Oxford: Blackwell Scientific Publications

[B14] Meyer M Schuster KD Schulz H Mohr M Piiper J Pulmonary diffusing capacities for nitric oxide and carbon monoxide determined by rebreathing in dogs J Appl Physiol 1990 68 6 2344 2357 2384415 10.1152/jappl.1990.68.6.2344

[B15] Rudski LG Lai WW Afilalo J Hua L Handschumacher MD Chandrasekaran K Solomon SD Louie EK Schiller NB Guidelines for the echocardiographic assessment of the right heart in adults: a report from the American Society of Echocardiography endorsed by the European Association of Echocardiography, a registered branch of the European Society of Cardiology, and the Canadian Society of Echocardiography J Am Soc Echocardiogr 2010 23 7 685 713 quiz 786–688 10.1016/j.echo.2010.05.010 20620859

[B16] Ozkan M Dweik RA Laskowski D Arroliga AC Erzurum SC High levels of nitric oxide in individuals with pulmonary hypertension receiving epoprostenol therapy Lung 2001 179 4 233 243 10.1007/s004080000064 11891614

[B17] Machado RF Londhe Nerkar MV Dweik RA Hammel J Janocha A Pyle J Laskowski D Jennings C Arroliga AC Erzurum SC Nitric oxide and pulmonary arterial pressures in pulmonary hypertension Free Radic Biol Med 2004 37 7 1010 1017 10.1016/j.freeradbiomed.2004.06.039 15336317

[B18] Agostoni P Bussotti M Cattadori G Margutti E Contini M Muratori M Marenzi G Fiorentini C Gas diffusion and alveolar-capillary unit in chronic heart failure Eur Heart J 2006 27 21 2538 2543 10.1093/eurheartj/ehl302 17028107

[B19] Tonelli AR Plana JC Heresi GA Dweik RA Prevalence and prognostic value of left ventricular diastolic dysfunction in idiopathic and heritable pulmonary arterial hypertension Chest 2012 141 6 1457 1465 10.1378/chest.11-1903 22207680 PMC3367485

[B20] Puwanant S Park M Popovic ZB Tang WH Farha S George D Sharp J Puntawangkoon J Loyd JE Erzurum SC Ventricular geometry, strain, and rotational mechanics in pulmonary hypertension Circulation 2010 121 2 259 266 10.1161/CIRCULATIONAHA.108.844340 20048214 PMC2846516

[B21] Agostoni P Cattadori G Bianchi M Wasserman K Exercise-induced pulmonary edema in heart failure Circulation 2003 108 21 2666 2671 10.1161/01.CIR.0000097115.61309.59 14581402

[B22] Jing ZC Xu XQ Badesch DB Jiang X Wu Y Liu JM Wang Y Pan L Li HP Pu JL Pulmonary function testing in patients with pulmonary arterial hypertension Respir Med 2009 103 8 1136 1142 10.1016/j.rmed.2009.03.009 19403296

[B23] Rastogi D Ngai P Barst RJ Koumbourlis AC Lower airway obstruction, bronchial hyperresponsiveness, and primary pulmonary hypertension in children Pediatr Pulmonol 2004 37 1 50 55 10.1002/ppul.10363 14679489

[B24] Spiekerkoetter E Fabel H Hoeper MM Effects of inhaled salbutamol in primary pulmonary hypertension Eur Respir J 2002 20 3 524 528 10.1183/09031936.02.02572001 12358324

[B25] Meyer FJ Ewert R Hoeper MM Olschewski H Behr J Winkler J Wilkens H Breuer C Kubler W Borst MM Peripheral airway obstruction in primary pulmonary hypertension Thorax 2002 57 6 473 476 10.1136/thorax.57.6.473 12037220 PMC1746348

[B26] O'Hagan AR Stillwell PC Arroliga A Airway responsiveness to inhaled albuterol in patients with pulmonary hypertension Clin Pediatr 1999 38 1 27 33 10.1177/000992289903800104 9924639

[B27] Price LC Wort SJ Perros F Dorfmuller P Huertas A Montani D Cohen-Kaminsky S Humbert M Inflammation in pulmonary arterial hypertension Chest 2012 141 1 210 221 10.1378/chest.11-0793 22215829

[B28] Heath D Yacoub M Lung mast cells in plexogenic pulmonary arteriopathy J Clin Pathol 1991 44 12 1003 1006 10.1136/jcp.44.12.1003 1791199 PMC494968

[B29] Fernandez-Bonetti P Lupi-Herrera E Martinez-Guerra ML Barrios R Seoane M Sandoval J Peripheral airways obstruction in idiopathic pulmonary artery hypertension (primary) Chest 1983 83 5 732 738 10.1378/chest.83.5.732 6839814

[B30] Said SI Hamidi SA Gonzalez Bosc L Asthma and pulmonary arterial hypertension: do they share a key mechanism of pathogenesis? Eur Respir J 2010 35 4 730 734 10.1183/09031936.00097109 20356986 PMC2963099

[B31] Daley E Emson C Guignabert C de Waal MR Louten J Kurup VP Hogaboam C Taraseviciene-Stewart L Voelkel NF Rabinovitch M Pulmonary arterial remodeling induced by a Th2 immune response J Exp Med 2008 205 2 361 372 10.1084/jem.20071008 18227220 PMC2271018

